# Modeling Disease Vector Occurrence When Detection Is Imperfect II: Drivers of Site-Occupancy by Synanthropic *Triatoma brasiliensis* in the Brazilian Northeast

**DOI:** 10.1371/journal.pntd.0002861

**Published:** 2014-05-08

**Authors:** Carolina Valença-Barbosa, Marli M. Lima, Otília Sarquis, Claudia M. Bezerra, Fernando Abad-Franch

**Affiliations:** 1 Chagas Disease Eco-epidemiology Laboratory, Instituto Oswaldo Cruz – Fiocruz, Rio de Janeiro, Brazil; 2 Secretaria Estadual de Saúde do Ceará, Fortaleza, Brazil; 3 Infectious Disease Ecology Laboratory, Instituto Leônidas e Maria Deane – Fiocruz, Manaus, Brazil; Liverpool School of Tropical Medicine, United Kingdom

## Abstract

**Background:**

Understanding the drivers of habitat selection by insect disease vectors is instrumental to the design and operation of rational control-surveillance systems. One pervasive yet often overlooked drawback of vector studies is that detection failures result in some sites being misclassified as uninfested; naïve infestation indices are therefore biased, and this can confound our view of vector habitat preferences. Here, we present an initial attempt at applying methods that explicitly account for imperfect detection to investigate the ecology of Chagas disease vectors in man-made environments.

**Methodology:**

We combined triplicate-sampling of individual ecotopes (*n* = 203) and site-occupancy models (SOMs) to test a suite of pre-specified hypotheses about habitat selection by *Triatoma brasiliensis*. SOM results were compared with those of standard generalized linear models (GLMs) that assume perfect detection even with single bug-searches.

**Principal Findings:**

*Triatoma brasiliensis* was strongly associated with key hosts (native rodents, goats/sheep and, to a lesser extent, fowl) in peridomestic environments; ecotope structure had, in comparison, small to negligible effects, although wooden ecotopes were slightly preferred. We found evidence of dwelling-level aggregation of infestation foci; when there was one such focus, same-dwelling ecotopes, whether houses or peridomestic structures, were more likely to become infested too. GLMs yielded negatively-biased covariate effect estimates and standard errors; both were, on average, about four times smaller than those derived from SOMs.

**Conclusions/Significance:**

Our results confirm substantial population-level ecological heterogeneity in *T. brasiliensis*. They also suggest that, at least in some sites, control of this species may benefit from peridomestic rodent control and changes in goat/sheep husbandry practices. Finally, our comparative analyses highlight the importance of accounting for the various sources of uncertainty inherent to vector studies, including imperfect detection. We anticipate that future research on infectious disease ecology will increasingly rely on approaches akin to those described here.

## Author Summary

Chagas disease prevention depends on the control of its insect vectors — large blood-sucking bugs called triatomines. One commonly neglected problem of vector studies is imperfect detection, whereby some sites are mistakenly classified as uninfested. We address this drawback by combining repeated sampling of ‘ecotopes’ (houses, fowl-houses, corrals, woodpiles, etc.) with models that accommodate detection failures to study habitat selection by a major vector, *Triatoma brasiliensis*. *Triatoma brasiliensis* was strongly associated with peridomestic rodents and goats/sheep. We found little support to the conventional view that *T. brasiliensis* prefers stone-like habitats; at least in our study setting, host availability was much more important than ecotope structure, and, overall, wooden habitats (timber piles or wood-fenced corrals) were slightly favored. The risk of infestation in any individual ecotope increased when another ecotope in the same dwelling was also infested; this increase was the same for houses and for peridomestic structures. These findings suggest that management of peridomestic animals, and especially rodents and goats, could help mitigate the risk of dwelling and house infestation by *T. brasiliensis*. In sum, our study demonstrates how relatively simple but sound sampling and analytical approaches can critically enhance our understanding of disease ecology by explicitly accounting for imperfect detection.

## Introduction

*Triatoma brasiliensis* is a member of the ‘brasiliensis species complex’, which includes the main domestic-peridomestic vectors of the Chagas disease parasite, *Trypanosoma cruzi*, in the Caatinga eco-region of northeastern Brazil [1]–[4]. Wild *T. brasiliensis* breed in rocky outcrops, usually in association with terrestrial rodents such as species of *Kerodon*, *Galea* or *Thrichomys* but opportunistically feeding also on other vertebrates [3], [5]–[8]. In addition, some *T. brasiliensis* populations have adapted to exploit man-made ecotopes [3]–[6], [8]–[11]. In particular, it has been suggested that this species preferentially infests stone-like man-made structures such as stone/mud walls or tile/brick piles [5], [6], [8], [9]. This is in agreement with the more general view that synanthropic triatomines tend to occupy man-made ecotopes structurally resembling their original, wild microhabitats [5], [6]. However, *T. brasiliensis* foci have been reported from a wide range of man-made structures [3]–[6], [8]–[11], suggesting that these obligate blood-feeders may simply select microhabitats where vertebrates are available to be fed upon [5], [6]. Recent findings show, in fact, that wild *T. brasiliensis* often infest shrubby cacti co-occupied by rodents in rock-free sedimentary lowlands where infestation/re-infestation of man-made structures, including woodpiles, is commonplace [11], [12].

With the aim of advancing our knowledge about the drivers of ecotope selection by *T. brasiliensis* in man-made environments, we studied the subspecies known as *T. brasiliensis brasiliensis*, the most strongly synanthropic within the complex [1], [2], [8]. A pervasive yet thus far overlooked problem in this kind of investigation is that detecting triatomine infestation foci can be perplexingly difficult, in particular when colonies are small and occupy structurally complex ecotopes; naïve infestation indices that disregard detection failures are therefore prone to negative bias, and this can confound ecological inference [13], [14]. Acknowledging this key drawback, we chose an analytical framework that makes use of repeated ecotope-sampling data to explicitly incorporate detection failures in model-based parameter estimation [15], [16]. We thus derived unbiased statistical estimates of (i) the probability that an ecotope is occupied by the vectors (‘site-occupancy’), (ii) the probability that bugs are detected in an occupied ecotope, and (iii) the effects of selected covariates on those probabilities – each with the corresponding measure of uncertainty [15], [16] (see also Text S1).

Using this approach, we tested a set of *a priori* hypotheses about the drivers of site-occupancy by *T. b. brasiliensis* (Table 1). Our focal hypothesis states that *T. b. brasiliensis* preferentially occupies rock-like (mineral) man-made ecotopes [3], [5]–[8], regardless of vertebrate host availability. Alternatively, we postulated that local *T. b. brasiliensis* populations may preferentially occupy ecotopes where key hosts are available [3], [5], [6], [9], [10], [17], regardless of ecotope structure. To test these core hypotheses and their main specific versions (see examples in Table 1), we combined hierarchical modeling and information-theoretic model ranking and averaging; this allowed us to make strong inferences about the vectors' microhabitat preferences [15], [16], [18]. Our results support a major role of key-host availability in habitat selection by our study *T. b. brasiliensis* population, and demonstrate how such a robust approach can be applied to the epidemiologically relevant scenario of dwelling infestation by disease vectors. This opens new possibilities for the investigation of pathogen transmission risk under realistic field conditions.

**Table 1:** Examples of site-occupancy models and associated hypotheses about ecotope infestation by *Triatoma b. brasiliensis*.

Occupancy structure*	Hypothesis•
Ψ(.) [‘null’ model]	Infestation is randomly distributed among ecotopes
Ψ(SDEc)	Infestation is simply a function of infestation in same-dwelling ecotopes
Ψ(SDEc,H)	Infestation is simply a function of the “domestic/peridomestic” dichotomy (after adjusting for same-dwelling ecotope infestation)†
Ψ(SDEc,M)	Bugs tightly associated with particular (stone-like) ecotopes
Ψ(SDEc,H,M)	Infestation is a function of the “domestic/peridomestic” dichotomy but, in the peridomestic area, it depends on the main structural traits of ecotopes
Ψ(SDEc,NR)	Bugs tightly associated with native rodents
Ψ(SDEc,NR,GS)	Infestation depends on the availability of native rodents and goats/sheep, which are the main hosts
Ψ(SDEc,NR,GS,F)	Infestation depends on the availability of some key hosts (native rodents, goats/sheep, and fowl), with humans, cattle/pigs and dogs/cats having little or no influence
Ψ(SDEc,H,NR,GS,F)	Infestation depends on the availability of key hosts including humans, with cattle/pigs and dogs/cats having little influence
Ψ(SDEc,M,NR,F)	Infestation driven by the structural traits of peridomestic ecotopes and the availability of native rodents and fowl
Ψ(SDEc,M,NR,GS)	Infestation driven by the structural traits of peridomestic ecotopes and the availability of native rodents and goats/sheep
Ψ(SDEc,H,M,NR,GS)	Infestation driven by both ecotope structural traits and the availability of two key hosts (native rodents and goats/sheep)
Ψ(SDEc,M,NR,GS,F)	Infestation driven by the structural traits of peridomestic ecotopes and the availability of three key hosts (native rodents, goats/sheep, and fowl)
Ψ(SDEc,H,M,NR,GS,F,CP,DC)	Infestation varies widely across ecotope structures and with host availability

*All models had a ‘*p*(v_1_)’ sampling-process (detection) structure (see main text).

• Each specific “hypothesis” can be interpreted as the conclusion that would be drawn if the associated model were unequivocally better supported by the data than any alternative model; particular interpretations would of course need to take estimates of effect size, and their sign and precision, into account.

† The last part of this statement, “(after adjusting for same-dwelling ecotope infestation)”, applies to all models in which the SDEc covariate appears along with further covariates.

Covariates: SDEc, Same-Dwelling Ecotope infestation; H, House; M, All_Mineral; NR, Native_Rodent; GS, Goat/Sheep; F, Fowl; CP, Cattle/Pig; DC, Dog/Cat (see main text for definitions and values).

## Methods

### Ethics statement

This study is part of long-term research on Chagas disease eco-epidemiology in the state of Ceará, Brazil, led by MML and approved by the Fiocruz Institutional Review Board (CEP/Fiocruz protocol 139/01), the Brazilian Environmental Agency (IBAMA/Sisbio protocol 14323-6), and the Fiocruz Committee for Animal Research (CEUA/Fiocruz protocol P59-12-2).

### Study setting

We selected a rural area of the state of Ceará where *T. b. brasiliensis* persistently re-infests human dwellings despite long-term chemical control efforts (CMB, unpublished). The study site (approx. 4°52′S, 37°52′W) lies within the core *T. b. brasiliensis* distribution area in the Caatinga eco-region [2], [4], and specifically in the sedimentary lowlands of the middle-lower Jaguaribe river basin. The area has a mixed landscape with crops (maize, beans, rice), some pastures, and patches of moderately well-preserved Caatinga xeric shrubland, where vegetation includes ‘catingueira’ (*Caesalpinia pyramidalis*) and other hardwood trees (*Licania* sp., *Auxemma* sp., *Aspidosperma* sp.), *Copernicia prunifera* palms, cacti (*Pilosocereus gounellei*, *Cereus jamacaru*), and thorny shrubs. Rocky outcrops were not observed in the study area [12]. The climate is hot and dry, with mean temperatures ∼23–33°C (absolute range, 16–38°C) and rainfall <850 mm/year with periodic severe droughts. Typical dwellings are scattered compounds with a house and a rather complex peridomestic area in which animals (mainly fowl, goats, pigs, dogs, and cattle) are reared and family goods stored.

### Sampling strategy

All dwellings in the area were visited and included in the survey unless householders were absent or refused to participate. Within each dwelling compound, all discrete ecotopes (the house plus all peridomestic structures, including storerooms, corrals, fowl-houses, kennels, pigsties, and piles of timber, bricks, stones or tiles) were searched for triatomines. We mimicked routine surveys performed by local health services in two important ways: first, bug searches were conducted by local vector control-surveillance staff using their own standard methods; second, each dwelling was visited once to determine infestation status, and a second visit was scheduled for about a week later to spray the dwelling with a pyrethroid insecticide. Differently from routine practice, a second bug-search was conducted during the second visit, prior to insecticide spraying, and yet another bug-search after each ecotope was sprayed – which might reveal hidden infestation foci due to the irritant and ‘knock-down’ effects that pyrethroids have on triatomines. All houses and peridomestic structures, irrespective of their observed infestation status, were therefore sprayed using standard methods and within the usual time-frame of routine vector control.

Several procedures were implemented to minimize the influence of each bug-search on subsequent searches. First, field teams were shifted in each visit so that they did not search the same ecotopes more than once, and each team was kept blind to the results of previous searches. Second, since all ecotopes were to be further searched and sprayed within a few days, field teams were instructed to stop searching in each individual ecotope as soon as one *T. brasiliensis* specimen was detected during the first and second visits, without collecting any bugs except if seen inside human residences. Operational constraints precluded the application of these procedures in a few cases; we noted these exceptions and, when feasible, used this information to control for their possible effects during data analysis (see below and Text S1).

The result of each bug-search was recorded separately, yielding a ‘detection history’ for each ecotope; for example, the detection history “001” indicates that in this ecotope no bugs were detected in the first and second bug-searches (the two “0”s), but at least one bug was detected in the third search (the last “1”). Assuming that the population in each ecotope was ‘closed’ (no local extinction or colonization) during the short sampling period, this allowed us to estimate bug-detection probabilities and correct for false-negative results [15], [16] (see also Text S1). Overall, 203 discrete ecotopes were surveyed in 32 dwelling compounds, for a total of 609 individual bug-searches; for each ecotope, we noted structural traits (type, building materials, size) and use by vertebrates (see below) on which triatomines could feed (number of individuals or, in the case of rodents, a score measuring the amount of feces present in the ecotope: from 0 if absent to 5 if extremely abundant [12], [19]). In addition, we recorded the time elapsed since each dwelling was last sprayed by professional vector-control staff.

### Data analysis

For the purposes of the present investigation, we only consider data on *T. b. brasiliensis*, which represented the vast majority of detections. We first conducted exploratory analyses using observed “presence/absence” naïve data – that is, without taking possible detection failures into account. To get an initial sense of the possible effects of our covariates (see below), we used bivariate null-hypothesis tests (Pearson's χ^2^) and unadjusted conditional maximum-likelihood odds ratios (ORs) with 95% exact confidence intervals (CIs); these analyses were conducted using OpenEpi 3.01 [20]. In a second stage, we used a modeling framework that explicitly accounts for detection failures (see Text S1) and combines maximum-likelihood and information theories to provide a measure of the relative support that different hypotheses find in the data [15], [16], [18]. In this approach, each specific hypothesis about site-occupancy (infestation) by *T. b. brasiliensis* is represented by a hierarchical model (see examples in Table 1), and Akaike's information criterion (AIC) and related metrics are used to rank the models [15], [16], [18]. We used the second-order version of AIC, AICc, and, after goodness-of-fit testing, the overdispersion-corrected version of AICc, quasi-AICc (QAICc; see below and refs. [18], [21]). The models use the logit link function to evaluate covariates on detection (denoted *p*) and/or on site-occupancy probabilities (denoted Ψ), so that slope coefficients (*β*s) and their variances are also estimated (see refs. [15], [16] and Text S1); the combination of model ranking and covariate 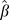
s is then used to make strong inferences about the hypotheses under consideration [15], [16], [18]. Site-occupancy modeling and related procedures were carried out using the program Presence 6.1 [22].

#### Null models and same-dwelling aggregation

**Figure 1:**
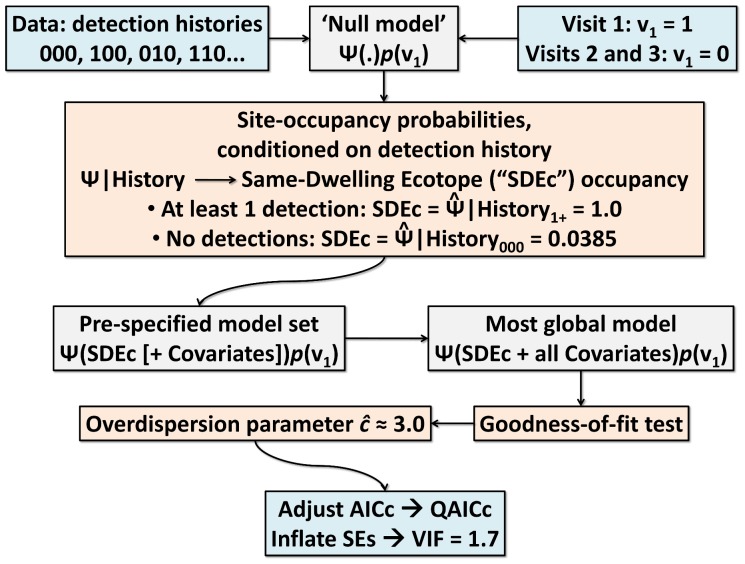
Ecology of synanthropic *Triatoma b. brasiliensis*: Flow chart illustrating the site-occupancy modeling strategy.

‘Null’ occupancy models represent the null hypothesis of randomly distributed site-occupancy (Table 1). After preliminary analyses, we considered three ‘null’ models: (i) a ‘totally null model’ with constant Ψ and *p*, represented by “Ψ(.)*p*(.)”; (ii) a ‘full-identity null model’ in which *p* is allowed to vary between the first, second, and third bug-searches, “Ψ(.)*p*(full)”; and (iii) a ‘first-visit (“v_1_”) null model’ in which *p* can differ between the first and the two subsequent bug-searches, “Ψ(.)*p*(v_1_)”. The ‘best’ (lowest-AICc) null model was then used to estimate site-occupancy probabilities, conditioned on detection history (“Ψ|History”) (Fig. 1). For each ecotope, this conditional probability is 1.0 when at least one bug was detected, but, acknowledging imperfect detection, non-zero values are estimated when no bugs were seen after three visits (History_000_). Because our ecotopes were clustered within households, we used these 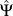
|History values to build a covariate describing, for each ecotope, infestation in other, same-dwelling ecotopes: “SDEc” = 1.0 if at least one same-dwelling ecotope was infested, and “SDEc” = 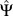
|History_000_ if bugs were not detected in any of the same-dwelling ecotopes after three visits. The “SDEc” covariate was included in all the rest of models (Fig. 1). For a similar covariate-based treatment of spatial dependencies in the context of site-occupancy modeling, see ref. [23].

#### Site-occupancy models

To test our focal hypothesis that site-occupancy by *T. b. brasiliensis* primarily depends upon *ecotope structure*, with a preference for stone-like materials, we built a covariate (“All_Mineral”) indexing whether each peridomestic ecotope was composed only of mineral materials (stones, bricks, tiles, dried mud, clay, concrete, or combinations thereof; covariate value 1) or was totally or partially composed of vegetal parts (value 0). Houses (covariate “House”) were considered separately because (i) we thought that vectors might be more likely to be noticed and killed by dwellers inside than around houses, which could confound the assessment of house-trait effects, and (ii) this allowed us to test the hypothesis that *T. b. brasiliensis* preferentially infests peridomestic structures rather than houses (see Table 1). Most houses had brick/adobe walls and tile roofs, and a few had some wooden parts.

The main alternative to the ecotope-structure hypothesis is the *host-availability* hypothesis. We defined “availability” in terms of whether there was any evidence that at least one host was making use of a particular ecotope. We defined six host categories: (i) rodents or their traces; after preliminary analyses, we considered native rodents only (covariate “Native_Rodent”), with rodent feces identified after ref. [19]; (ii) goats/sheep or their traces (“Goat/Sheep”); (iii) fowl (mainly chickens) or their traces (“Fowl”); (iv) dogs/cats (“Dog/Cat”); (v) cattle/pigs (“Cattle/Pig”); and (vi) humans (“Human”). Note that, since humans were ‘available’ in all houses, covariates “House” and “Human” had the same 0/1 values for each ecotope.

With these covariates, we defined a ‘core’ set of 31 additive hierarchical models, each representing a plausible *a priori* hypothesis about the drivers of site-occupancy by *T. b. brasiliensis* (Tables 1 and S1). All models included the “SDEc” term derived from Ψ|History and, after ‘null’ model analyses (see below), a detection covariate distinguishing the first from the two subsequent surveys (“v_1_”) (Fig. 1).

We tested for goodness-of-fit (GOF) in the most global model in our *a priori* model set (model M19 in Table S1) using parametric bootstrapping [21] with 10,000 pseudo-replicates. This test also provides an estimate (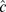) of overdispersion; 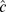 values≠1.0 indicate extra-binomial variation [21] and should be used to adjust standard errors (SEs) and to correct AICc scores (using QAICc instead) and model weights [18], [21]. The GOF test suggested moderate overdispersion (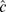 = 3.47); since, however, we expect 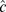 to be biased up by ∼12–14% [24], we used 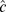 = 3.0 as a simpler estimate, and √3.0 = 1.7 as our variance inflation factor (VIF) (Fig. 1) [18], [21], [24], [25]. Weighted mean effect-sizes (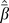
s) were estimated for each covariate appearing in the subset of models whose Akaike weights (*w*_i_) summed to ∼0.95 [18]. Model-averaged estimates are the sum of model-specific 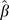
s times model-specific *w*_i_, with *w*_i_ renormalized to sum to 1.0 across models with the covariate under consideration [18]. To be consistent with our AIC-based approach to model assessment (see ref. [26]), we present 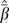
s with approximate 85% confidence intervals (CIs) based on unconditional, VIF-inflated SEs [18], [21].

We finally tested whether well-supported models (ΔQAICc<2.0) including host-availability covariates were improved to any extent by using standardized host numbers (‘feces score’ for rodents) instead of host presence/absence data. We also evaluated a model including the “All_Mineral” covariate and an estimate of host biomass in each ecotope – a weighted sum with weights set as follows: rodent ‘feces score’, 0.2; fowl, 1.0; cat, 1.5; dog, 5.0; goat/sheep, 10; human, 10; pig, 20; and cattle, 100.

#### Generalized linear models

We investigated the consequences of ignoring sampling-process and model-selection uncertainty by re-fitting the subset of site-occupancy models with ΔQAICc<2.0 using generalized linear models (GLMs) with a binomial distribution and the logit link function, as implemented in JMP 9.0 (SAS Institute, Cary, NC, USA). The binomial response was 1 when at least one bug was detected (i) in an individual bug-search during the first, second, or third visits (which were modeled separately to mimic typical single-visit surveys) or (ii) in at least one of the three bug-searches (i.e., with the three search results combined); otherwise, the response value was 0. Similarly, the “SDEc” covariate took a value of 1 if at least one bug was detected in at least one same-dwelling ecotope and 0 otherwise; this covariate was derived for each visit separately and for all three visits combined. We tested our GLMs for lack-of-fit and overdispersion with the methods implemented in JMP 9.0. These GLMs make the implicit assumption that bug searches have 100% sensitivity (separately for the visit-specific GLMs and with all visits combined for the last GLM), and therefore that non-detection equals true absence: in other words, they treat observed, naïve infestation data as if they were a fully reliable representation of reality [13]–[16]. With this comparison, we investigated whether and to what extent different modeling approaches could lead to different conclusions about the same biological problem – habitat selection by *T. b. brasiliensis*. Our comparative appraisal focuses on covariate effect-size (*β*) estimates and their SEs derived from site-occupancy models and GLMs.

## Results

### Descriptive results and exploratory analyses

We surveyed 203 man-made ecotopes in 32 dwelling compounds (a house plus its peridomestic structures), none of which had been professionally sprayed with insecticides within the 24 months before fieldwork; the mean number of ecotopes per dwelling was 6.3 (range 2–12). Infestation by *T. b. brasiliensis* was detected in 66 ecotopes, so the ecotope-level naïve infestation index was II_ecotope_ = 32.5%. At the dwelling scale, only 11 out of 32 compounds appeared to be free of infestation (II_dwelling_ = 65.6%). Bugs were found inside four houses (4/32; II_domestic_ = 12.5%), which therefore seemed to be at a substantially lower risk of infestation than peridomestic ecotopes (62/171 infested: II_peridomestic_ = 36.3%; Table 2). Among these 171 peridomestic ecotopes, infestation was observed less frequently in all-mineral ecotopes (27.3%) than in those with vegetal parts (41.9%), but this difference was marginally non-significant (Table 2). Conversely, all-vegetal peridomestic ecotopes had higher odds of infestation than those with at least some mineral parts, but, again, this difference did not appear to be significant (Table 2). Further details about observed infestation patterns in different ecotope types are presented in Table 2.

**Table 2:** Observed ecotope-level infestation by *Triatoma b. brasiliensis* and ecotope characteristics: descriptive results and bivariate exploratory analyses.

Ecotope trait		Observed infestation*		II	χ^2^	*P*	OR	CI
		Yes	No					
Overall (*n* = 203)								
House	Yes	4	28	12.50	6.934	0.0085	0.25	0.07, 0.71
	No	62	109	36.26			Ref.	
Goat/sheep corral	Yes	14	11	56.00	7.168	0.0074	3.07	1.30, 7.39
	No	52	126	29.21			Ref.	
Fowl-house	Yes	11	18	37.93	0.453	0.5010	1.32	0.57, 2.98
	No	55	119	31.61			Ref.	
Pigsty	Yes	4	15	21.05	1.255	0.2636	0.53	0.15, 1.58
	No	62	122	33.70			Ref.	
Cattle corral	Yes	2	4	33.33	0.002	0.9652	1.04	0.13, 6.01
	No	64	133	32.49			Ref.	
Storeroom	Yes	2	8	20.00	0.751	0.3863	0.51	0.07, 2.26
	No	64	129	33.16			Ref.	
Tile/brick pile	Yes	16	41	28.07	0.713	0.3985	0.75	0.38, 1.46
	No	50	96	34.25			Ref.	
Timber pile	Yes	13	10	56.52	6.815	0.0090	3.10	1.27, 7.73
	No	53	127	29.44			Ref.	
Peridomestic (*n* = 171)								
All mineral	Yes	18	48	27.27	3.754	0.0527	0.52	0.26, 1.01
	No	44	61	41.90			Ref.	
All vegetal	Yes	23	26	46.94	3.391	0.0656	1.88	0.95, 3.72
	No	39	83	31.97			Ref.	

*Number of ecotopes in each category.

II, WHO infestation index (%) [27]; χ^2^, Pearson's chi-square statistic; *P*, two-tailed *P*-value; OR, unadjusted conditional maximum-likelihood odds ratio; CI, exact 95% confidence interval; Ref., reference level.

Evidence of occupation by rodents was found in 24 ecotopes; feces were identified [19] as those of native species (mainly *Galea* sp., but perhaps *Thrichomys* sp. as well) in 21 ecotopes, and as those of non-native *Rattus* sp. and *Mus* sp. in the remaining three. Infestation by *T. b. brasiliensis* was very frequent in ecotopes occupied by rodents in general (75.0%) and, especially, by native rodents (85.7%; Table 3). Infestation was also frequent (52.2%) in ecotopes occupied by goats/sheep, whereas no association of the bugs with other domestic animals was apparent (Table 3). As noted above, the comparison of houses *vs.* peridomestic structures applies also to the availability of humans, because every house was occupied by at least one person.

**Table 3:** Observed ecotope-level infestation by *Triatoma b. brasiliensis* and vertebrate host availability: descriptive results and bivariate exploratory analyses.

Host presence		Observed infestation*		II	χ^2^	*P*	OR	CI
		Yes	No					
Rodents (all)	Yes	18	6	75.00	22.393	<0.0001	8.09	3.11, 23.44
	No	48	131	26.82			Ref.	
Rodents (native)	Yes	18	3	85.71	30.215	<0.0001	16.49	5.04, 72.97
	No	48	134	26.37			Ref.	
Goats/Sheep	Yes	24	22	52.17	10.479	0.0012	2.97	1.50, 5.91
	No	42	115	26.75			Ref.	
Fowl	Yes	12	18	40.00	0.900	0.3429	1.47	0.64, 3.27
	No	54	119	31.21			Ref.	
Dogs/Cats	Yes	3	18	14.29	3.546	0.0597	0.32	0.07, 1.03
	No	63	119	34.62			Ref.	
Cattle/Pigs	Yes	6	17	26.09	0.488	0.4848	0.71	0.24, 1.84
	No	60	120	33.33			Ref.	
Humans	Yes	4	28	12.50	6.934	0.0085	0.25	0.07, 0.71
	No	62	109	36.26			Ref.	

*Number of ecotopes in each category.

II, WHO infestation index (%) [27]; χ^2^, Pearson's chi-square statistic; *P*, two-tailed *P*-value; OR, unadjusted conditional maximum-likelihood odds ratio; CI, exact 95% confidence interval; Ref., reference level.

### Site-occupancy models

Null-model analyses suggested that the bug-detection process was better described by distinguishing the first from the two subsequent bug-searches (model Ψ(.)*p*(v_1_); AICc = 505.07) than by either assuming constant detection probabilities (ΔAICc = 10.48) or allowing *p* to differ among all three bug-searches (ΔAICc = 1.97). Our ‘best’ null model estimated higher detection probabilities for the first bug-search (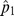 = 0.730, SE = 0.061) than for the second and third bug-searches (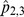 = 0.477, SE = 0.046); detection probabilities were therefore high for all visits combined (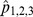 = 0.926), and the null model-based estimate of mean site-occupancy (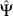 = 0.351, SE = 0.037) was barely above the observed naïve infestation index after three bug searches (II_ecotope_ = 0.325). This null model estimated Ψ|History_000_ at 0.0385, suggesting that an individual ecotope in which three bug-searches yielded no detection still had a chance of about 3.85% of being infested. These values were used to build a covariate [23] describing, for each ecotope, infestation in other, same-dwelling ecotopes: “SDEc” = 1.0 if at least one bug was detected in at least one same-dwelling ecotope, and 0.0385 otherwise. A “v_1_” sampling-process covariate and the “SDEc” occupancy covariate were included in all our subsequent models (Fig. 1; see Table S1 for the complete set of *a priori* site-occupancy models). We tested for GOF in the most global model and used 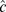 = 3.00 to account for overdispersion [18], [21], [24], [25] (Fig. 1).

Table 4 presents the subset of models with Σ*w*_i_ = 0.956. The top- and the second-ranking models include, besides “SDEc”, only host-availability covariates on Ψ; both explain the data about equally well (ΔQAICc<1.0), and differ in the inclusion or exclusion of the “Fowl” covariate (Table 4). Model M1 estimated ecotope infestation probabilities as ranging from 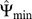 = 0.051 (SE = 0.026) in ecotopes with no same-dwelling ecotopes found infested and no evidence of native rodent, goat/sheep, or fowl availability to 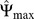 = 0.989 (SE = 0.020) in a wooden hen-house co-occupied by 10 chickens and a colony of native rodents in a dwelling where other ecotopes were also infested. Models M3 and M4 include the “All_Mineral” ecotope-structure covariate. Both are less than 2.0 QAICc units from the top-ranked model, yet they do not explain the data any better that the simpler M1 and M2 models. Models M5 to M9 (ΔQAICc<4.0) include the “House” covariate; “Cattle/Pig” and “Dog/Cat” covariates only appear in models with ΔQAICc>4.0 (Tables 4 and S1). Of note, our ‘null’ model was >13.0 QAICc units from the top-ranking model (Table S1), suggesting that the covariates we selected capture important aspects of habitat selection by *T. b. brasiliensis*. A model with just the “SDEc” covariate had a ΔQAICc>10.0, indicating that infestation did not depend only on dwelling-level aggregation (Tables 1 and S1; details not shown).

**Table 4:** The subset of models with ΣAkaike weights ≈0.95, with models ranked by their QAICc scores.

Model	Occupancy (Ψ) and detection (*p*) covariates	QAICc	ΔQAICc	*w*_i_	Likelihood	Deviance
M1	Ψ(SDEc,Native_Rodent,Goat/Sheep,Fowl)*p*(v_1_)	159.07	0	0.196	1	433.50
M2	Ψ(SDEc,Native_Rodent,Goat/Sheep)*p*(v_1_)	159.85	0.78	0.133	0.677	442.25
M3	Ψ(SDEc,All_Mineral,Native_Rodent,Goat/Sheep,Fowl)*p*(v_1_)	159.89	0.82	0.130	0.664	429.44
M4	Ψ(SDEc,All_Mineral,Native_Rodent,Goat/Sheep)*p*(v_1_)	160.27	1.20	0.108	0.549	437.08
M5	Ψ(SDEc,House,Native_Rodent,Goat/Sheep,Fowl)*p*(v_1_)	161.23	2.16	0.067	0.340	433.47
M6	Ψ(SDEc,House,Native_Rodent,Goat/Sheep)*p*(v_1_)	161.79	2.72	0.050	0.257	441.64
M7	Ψ(SDEc,House,Native_Rodent,Goat/Sheep,Fowl,Cattle/Pig)*p*(v_1_)	161.79	2.72	0.050	0.257	428.56
M8	Ψ(SDEc,House,All_Mineral,Native_Rodent,Goat/Sheep)*p*(v_1_)	161.88	2.81	0.048	0.245	435.42
M9	Ψ(SDEc,House,All_Mineral,Native_Rodent,Goat/Sheep,Fowl)*p*(v_1_)	162.05	2.98	0.044	0.225	429.35
M10	Ψ(SDEc,Native_Rodent)*p*(v_1_)	163.23	4.16	0.025	0.125	458.79
M11	Ψ(SDEc,House,All_Mineral,Native_Rodent,Goat/Sheep,Fowl,Cattle/Pig)*p*(v_1_)	163.28	4.21	0.024	0.122	426.41
M12	Ψ(SDEc,House,Native_Rodent,Goat/Sheep,Cattle/Pig)*p*(v_1_)	163.78	4.71	0.019	0.095	441.11
M13	Ψ(SDEc,House,Native_Rodent,Goat/Sheep,Fowl,Cattle/Pig,Dog/Cat)*p*(v_1_)	163.91	4.84	0.018	0.089	428.29
M14	Ψ(SDEc,House,All_Mineral,Native_Rodent,Goat/Sheep,Cattle/Pig)*p*(v_1_)	164.06	4.99	0.016	0.083	435.38
M15	Ψ(SDEc,House,Native_Rodent)*p*(v_1_)	164.21	5.14	0.015	0.077	455.33
M16	Ψ(SDEc,All_Mineral,Native_Rodent)*p*(v_1_)	164.59	5.52	0.012	0.063	456.48

QAICc, quasi-AICc (AICc, Akaike information criterion corrected for sample size); ΔQAICc, difference in QAICc between each model and the lowest-QAICc (top-ranking) model; *w*_i_, Akaike model weight; Likelihood, likelihood of each model, given the data (or relative strength of evidence for each model). SDEc, Same-Dwelling Ecotope infestation. See main text for the definitions and values of covariates, and ref. [18] for formulae and details on QAICc and related metrics.

**Figure 2:**
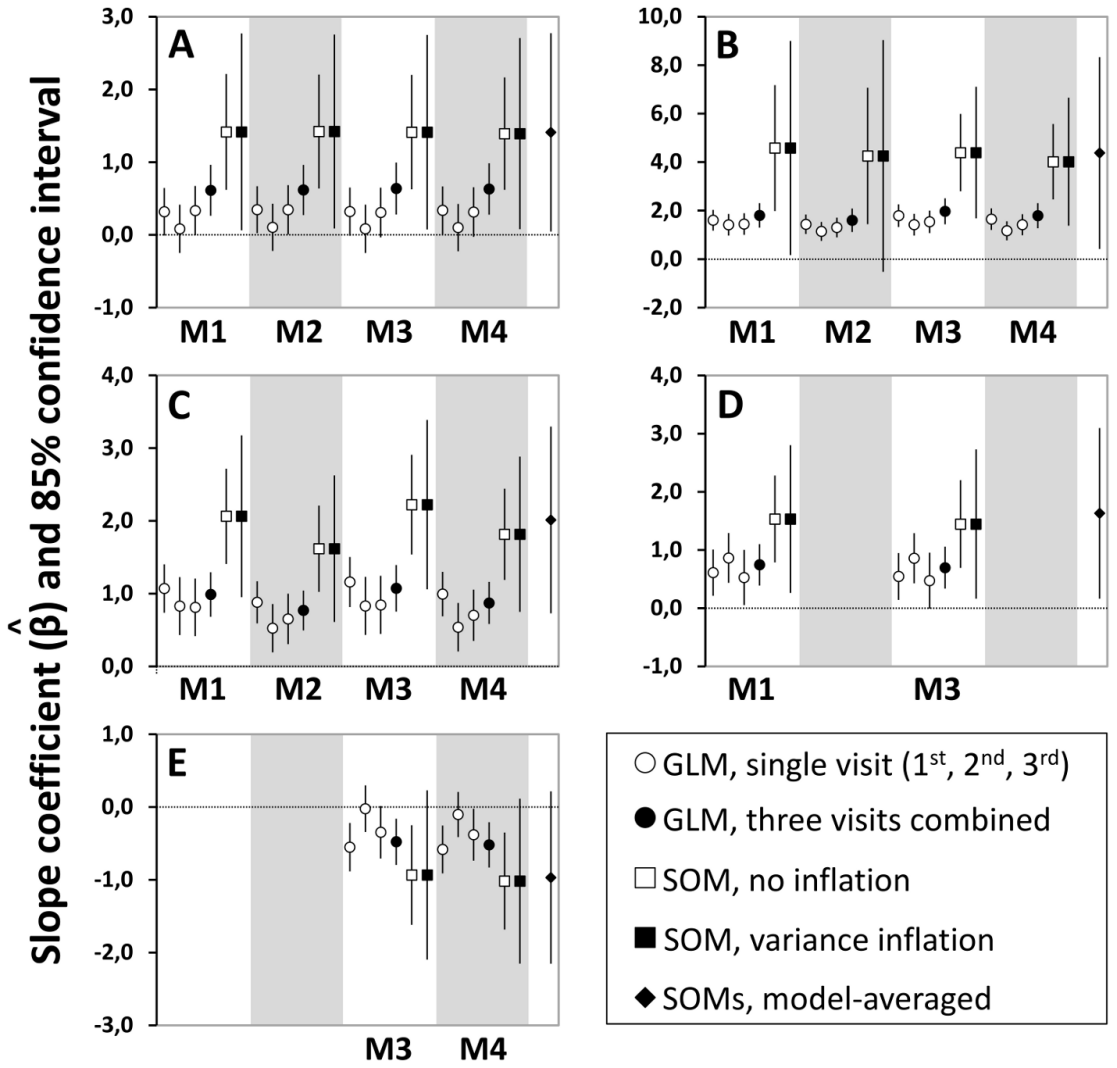
Main drivers of site-occupancy by synanthropic *Triatoma b. brasiliensis* in the Jaguaribe valley, Ceará, Brazil. Effect-size (*β*) estimates derived from the four top-ranked site-occupancy models (SOMs; models M1 to M4 in Table 4) and from generalized linear models (GLMs) with the same structure but assuming perfect detection and using single-visit data or data from three visits combined. Covariates: A, same-dwelling ecotope infestation (“SDEc” covariate); B, native rodents or their traces; C, goats/sheep or their traces; D, fowl or their traces; E, all-mineral ecotope. Confidence intervals (CIs) were estimated with and without variance inflation, as indicated. For model-averaged estimates (last estimate in each panel), inflated unconditional SEs [18] were used to construct approximate CIs. The effect of a covariate on ecotope infestation is considered indistinguishable from zero when the CI crosses the dotted line at *β* = 0.0. See main text for further details.

We next used the subset of models with Σ*w*_i_≈0.95 (Table 4) to derive model-averaged estimates of covariate *β*s [18], [26]. The results show a positive effect of the “SDEc” covariate (Table 5, Fig. 2); interestingly, the slope coefficient for the structure covariate “All_Mineral” was negative, yet the effect was relatively small and the CI included zero (Table 5, Fig. 2). The 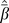 coefficient of the “House/Human” covariate was also negative, but the effect was very weak and the CI again included zero (Table 5).

**Table 5:** Model-averaged slope coefficient estimates from the subset of models with Akaike weights summing to ≈0.95 (see Table 4).

Covariate	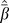	SE	CI_lower_	CI_upper_
Same-dwelling ecotope	1.41	0.95	0.05	2.77
Native rodent	4.38	2.75	0.42	8.33
Goat/Sheep	2.01	0.89	0.73	3.30
Fowl	1.63	1.02	0.17	3.10
All mineral	−0.97	0.82	−2.15	0.21
House/Human	−0.08	1.33	−2.00	1.84
Cattle/Pig	1.23	1.42	−0.81	3.27
Dog/Cat	0.58	1.95	−2.23	3.38

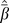
, model-averaged slope coefficient point estimate; SE, inflated unconditional standard error [18]; CIlower and CIupper, lower and upper limits of the approximate 85% confidence interval [26].

As expected, host-availability covariates (excluding “Human”) all had positive slope coefficients (Table 5). The “Native_Rodent” covariate had a strong positive effect on site-occupancy; uncertainty about this estimate seemed considerable (Table 5, Fig. 2), but this merely reflects that Ψ estimates were near the boundary (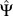
≈1.0) in rodent-occupied ecotopes. The presence of goats/sheep and, to a lesser extent, fowl was also associated with higher infestation odds, whereas the effects of dogs/cats and cattle/pigs were indistinguishable from zero (Fig. 2, Table 5).

The unexpected negative effect of the “All_Mineral” covariate prompted us to test, *a posteriori*, whether a “Some_Vegetal” covariate distinguishing peridomestic ecotopes with at least some vegetal parts from all-mineral and house ecotopes could help explain the data more parsimoniously. For this, we added the “Some_Vegetal” covariate to our two top-ranking models (Table 4). One of these new models (with the same host-availability covariates as model M2) ranked second, and estimated a positive effect of the “Some_Vegetal” covariate (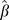 = 1.01, SE = 0.40). This model lumps all-mineral and house ecotopes into a single class with lower infestation risk than peridomestic ecotopes with vegetal parts in their structure.

Models using quantitative measures of host availability in each ecotope all performed substantially worse than their simpler, presence/absence counterparts (ΔQAICc>9.0); we therefore do not discuss them any further. Similarly, a model with a host biomass estimate and the “All_Mineral” covariate had essentially no support from the data (ΔQAICc≈14.0; details not shown).

### Generalized linear models

**Figure 3:**
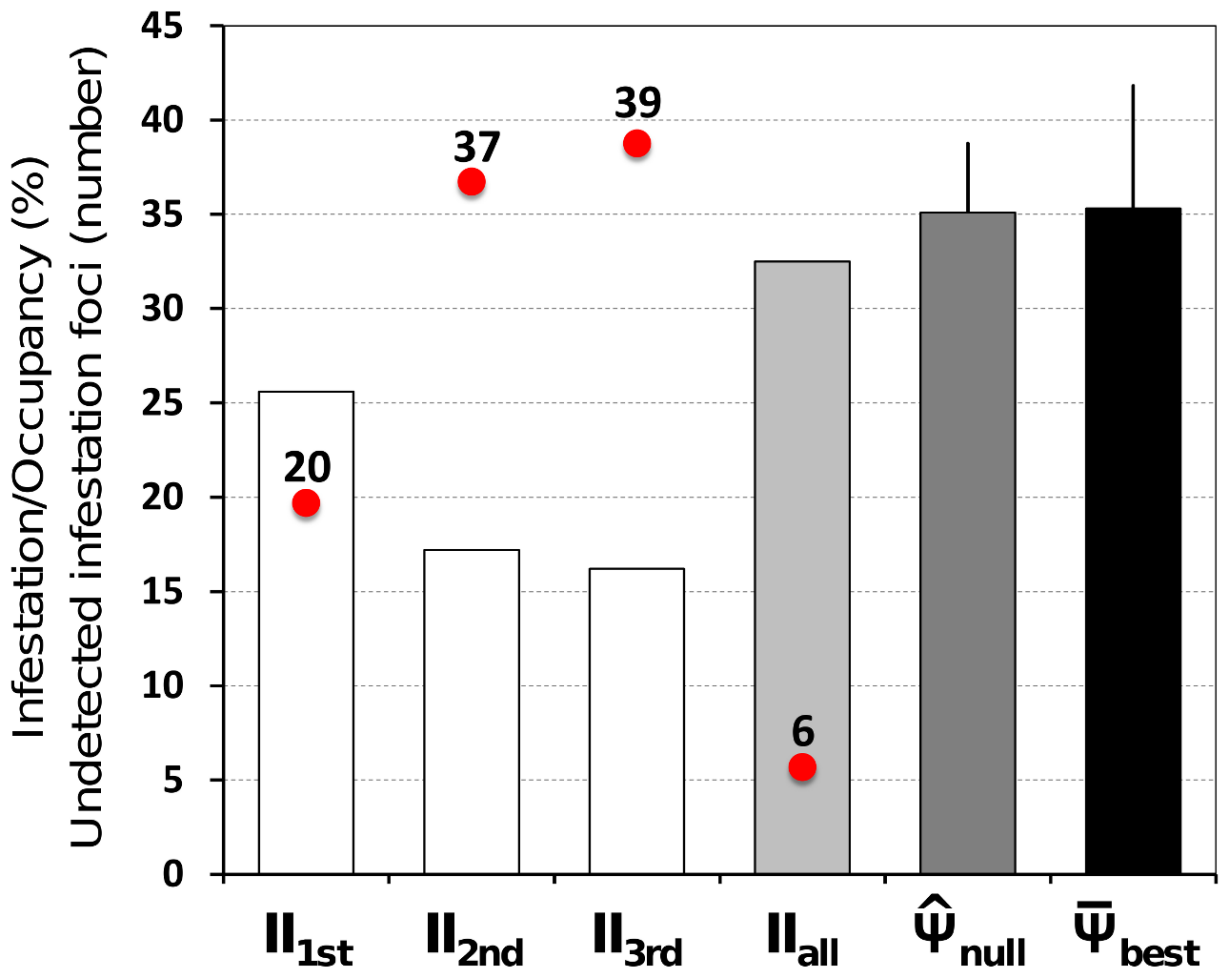
Ecotope infestation by synanthropic *Triatoma b. brasiliensis* in the Jaguaribe valley, Ceará, Brazil. Naïve infestation indices and site-occupancy estimates (bars): II, naïve infestation index from results of each single visit (II_1st_ to II_3rd_) and all visits combined (II_all_); 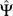
_null_, site-occupancy estimate (error bar, SE) derived from the ‘null model’ Ψ(.)*p*(v_1_) (see main text); 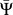
_best_, site-occupancy estimate derived from the ‘best’ model (M1; see main text and Table 4); this estimate is the mean of 203 ecotope-specific estimates derived from this model, and the error bar is the mean SE. Red circles show the estimated number of infestation foci that went undetected during single-visit bug-searches and all visits combined (rounded to the nearest integer).

Finally, we compared the results of our site-occupancy models with those of standard logit-binomial GLMs in which detection probabilities, conditioned on occurrence, are assumed to be *p* = 1.0 [13], [14]. Figure 2 shows the results of these comparisons, which involved the four model specifications (M1–M4, Table 4) with ΔQAICc<2.0 as well as the results of individual bug-searches separately and combined. Overall, we found that GLMs substantially underestimated both effect-sizes and their variances. Although the signs of point estimates did not change and effect-size bias was somewhat reduced by using the combined results of three bug-searches, qualitatively different conclusions could be drawn from the results of different modeling approaches. For example, the positive effect of the “SDEc” covariate appeared as indistinguishable from zero in most GLMs analyzing the results of single bug-searches (Fig. 2), and the effect of the “All_Mineral” covariate shifted from negative to indistinguishable from zero depending on the data used to fit the models (Fig. 2). The most striking finding was perhaps the gross overstatement of the precision of parameter estimates derived from GLMs – none of which, in addition, appeared to suffer from lack of fit or overdispersion judging by the output of JMP 9.0 (all 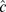
≈1.0; details not shown). GLM-derived *β* estimates were, on average, 4.2 (1.7–40.4) times smaller than those derived from site-occupancy models with the same structure. Similarly, GLM-derived SEs were from 2.1 to 5.8 times smaller than non-inflated SEs, and from 3.6 to 9.9 (mean, 4.6) times smaller than VIF-inflated SEs from site-occupancy models with the same covariates. Naïve ecotope-level infestation indices [27] derived from each single bug-search were II_1st_ = 25.6%, II_2nd_ = 17.2%, and II_3rd_ = 16.2%; when the results of all three visits were combined, this index rose, as mentioned above, to II_1,2,3_ = 32.5% (Fig. 3). Occupancy estimates from our ‘best’ model (M1) suggest that ∼20 to ∼39 infestation foci went undetected during single bug-searches (Fig. 3).

## Discussion

We have presented the first attempt to investigate the drivers of habitat selection by synanthropic triatomines using site-occupancy models that explicitly incorporate the imperfections of the sampling process [14]–[16]. We highlight three major findings. First, the *T. b. brasiliensis* population we studied clearly selects ecotopes that are occupied by some key vertebrate hosts, particularly native rodents and goats/sheep, irrespective of ecotope structure. Second, we found little support for the idea that this vector preferentially occupies peridomestic structures; if the right hosts are available and there is at least one infestation focus in a dwelling, the probabilities that the house or any other ecotope within that dwelling will become infested are about the same. And, third, standard analytic approaches that disregard detection failures (and other sources of uncertainty) can yield negatively-biased effect-size estimates with overly narrow confidence intervals; this can confound our view of the vectors' ecology and thus hinder the development, implementation, and assessment of effective control-surveillance strategies [13], [14], [28], [29] (see also Text S1).

### Modeling sampling-process uncertainty: Hurdles and caveats

Our repeated-sampling approach to addressing sampling-process uncertainty comes with some caveats. For example, we had to reduce the spatial scope of fieldwork to perform triplicate searches with the available resources [30]. This limited the breadth of inference, increased uncertainty about some estimates, and likely induced dependencies among same-dwelling ecotopes [11], [31], [32]. This latter possibility was accounted for by including a same-dwelling ecotope (“SDEc”) infestation covariate; this allowed us to quantify the effects of dwelling-level aggregation and to estimate adjusted *β*s for the ecotope-structure and host-availability covariates of focal interest [23].

The logistics of triplicate bug-searches can be quite complex and may result in further dependencies – this time among the serial searches performed in each ecotope. We took several precautions to minimize the effects of this potential problem (see **Methods**); however, applying all those measures was not fully feasible in every instance, and we suspect that some of our results reflect this difficulty. For example, we expected the third, post-insecticide-spraying bug-search to be more sensitive than the first and second searches, yet sensitivity was clearly higher in the first visit. We modeled this heterogeneity with our “v_1_” sampling covariate, but the finding still calls for an explanation. We suspect that disturbance produced during the first bug-search resulted in bugs hiding better during subsequent searches. Within houses, small infestation foci might have become depleted as bugs were collected during early searches; however, detection histories in the four infested houses (111, 101, 001, and 100) show that this could have been the case in just one of them. Overall, the low vagility of triatomines, particularly wingless nymphs, virtually ensured ecotope-level population ‘closure’ over the ∼7–10 day sampling period. The fact that GOF testing detected moderate overdispersion suggests, in any case, that there were further, un-modeled sources of sampling-process heterogeneity [21]. To address this issue, we investigated other factors that could possibly affect sampling outcomes (see Text S1), but none of them improved QAICc scores or model fit (details not shown). We therefore present our main models with the “v_1_” covariate on *p* and with AIC-related metrics and variance estimates corrected for overdispersion [18], [21], [24], [25].

Even with these and other, more general caveats (such as the cross-sectional nature of the survey or the rather limited sample size), we believe that our approach of incorporating sampling-process and model-selection uncertainty into the analyses represents a major improvement over traditional treatments of triatomine population ecology data [14], [28]. We note that while multi-model inference has been used in a few studies similar to ours [32]–[34], the formal treatment of detection failures has hardly entered the vector ecology literature thus far [14], [28], [29], [35], [36]. Yet, as discussed below, standard approaches can yield biased effect-size estimates and SEs; site-occupancy models may provide a more reliable and realistic picture of key population parameters and their environmental correlates [15], [16].

### Drivers of habitat selection by synanthropic *T. b. brasiliensis* populations

Our main results conflict with the conventional view that *T. b. brasiliensis* preferentially occupies man-made ecotopes that structurally resemble their natural, stony microhabitats [5], [6], [9]. Instead, model ranking and slope coefficient estimation both indicated that our study population tends to infest ecotopes where some key vertebrate hosts are available; ecotope-structure covariates had, in comparison, small to negligible effects on site-occupancy – and stone-like ecotopes were, if anything, at lower risk of infestation than those with vegetal parts (Table 5, Fig. 2). *A posteriori* analysis of a “Some_Vegetal” covariate indeed suggested a preference of local *T. b. brasiliensis* for vegetal ecotopes.

Notwithstanding this latter finding, our results indicate that the *T. b. brasiliensis* population we studied is tightly associated with native rodents, mainly *Galea* sp., which thrive around rural houses in the region. As already shown for other *T. brasiliensis* populations, goats/sheep are also major hosts [8], [9], [37]. Domestic fowl may have some secondary importance, and dogs/cats and cattle/pigs do not seem to play any significant role as resource-providers for local *T. b. brasiliensis* (Tables 3, 5, and S1). The fact that models with quantitative measures of host availability, including host biomass, explained the data much worse than host presence/absence models suggests that site-occupancy depended more on *which* hosts were available than on the amount of resources; a few *Galea* specimens dwelling in a timber pile may hence be of much higher value for our *T. b. brasiliensis* population than a cow weighting half a ton.

These results are in line with other reports from sites that, like our study area, lie within sedimentary Caatinga lowlands where rocky outcrops are rare or absent. There, synanthropic *T. b. brasiliensis* often infest timber piles [9], [11] and wild populations are common in shrubby cacti co-occupied by native rodents [12]. Together with previous findings on synanthropic and rock-dwelling *T. b. brasiliensis* [3]–[6], [7]–[11], [17], this confirms substantial ecological heterogeneity in this subspecies and suggests that such heterogeneity may be structured, with different habitat-selection trends in lowland (preferentially occupying vegetal ecotopes including cacti) *vs.* rocky-outcrop populations (preferentially associated with mineral ecotopes) [3]–[6], [8], [11], [12].

From a wider perspective, association with key hosts in structurally diverse ecotopes across landscapes and environments may indicate that *T. b. brasiliensis* ‘follows’ those key hosts as they colonize new microhabitats – and, particularly, that it originally followed terrestrial native rodents such as *Kerodon*, *Galea* or *Thrichomys* as they adapted to locally-available natural ecotopes: rocky outcrops in some areas, shrubby cacti in others. In our study setting, association with goats and fowl seemed secondary to the adaptation of native rodents and their allied triatomines to man-made ecotopes. Opportunistic exploitation of alternative hosts in wild habitats has also been suggested by bloodmeal analyses [7], [8], albeit the methods used to identify blood sources all have their drawbacks (see refs. [6], [38], [39]). Perhaps one further sign of an ancestral association with terrestrial rodents is that wild *T. brasiliensis* do not seem to occupy arboreal Caatinga habitats such as hardwood trees, where *T. pseudomaculata* occurs, or *Copernicia* palms, often infested by *Rhodnius nasutus* [12], [17], [40]. We note, finally, that a model with the same structure as our top-ranking model but using ‘all rodents’ instead of just native rodents performed relatively poorly (ΔQAICc = 3.32; details not shown), again suggesting a tight association of *T. b. brasiliensis* with *Galea*/*Thrichomys* in our study area.

Our exploratory analyses indicated, in agreement with previous studies [4], [8]–[11], [17], that *T. b. brasiliensis* preferentially infests peridomestic ecotopes (Table 2). Site-occupancy models, however, provided little evidence of any effect of the domestic/peridomestic dichotomy, as represented by the “House” covariate, on infestation odds (Tables 1 and 5); instead, they suggest that house infestation may more heavily depend on infestation of same-dwelling peridomestic ecotopes, which in turn depends on host availability. Thus, the apparent negative effect of houses seen in bivariate analyses (Table 2) became indistinguishable from zero when adjusted for same-dwelling ecotope infestation and host availability (Table 5). This underscores the importance of controlling peridomestic infestation foci to effectively protect people [3], [8], [10], [17], and, together with evidence that host availability is more important that ecotope structure, suggests that management of peridomestic animals could synergize the effects of insecticide spraying for the long-term control of synanthropic *T. b. brasiliensis*. Management strategies could include rodent control (e.g., by limiting the availability of suitable refuges, such as large timber or tile/brick piles, near houses) and changes in goat/sheep husbandry practices (e.g., by building corrals farther from houses and perhaps simplifying their structure [33]).

### Imperfect detection and bias in vector studies

To investigate whether and to what extent our more laborious approach may enhance triatomine ecology studies, we compared the results of site-occupancy models and standard GLMs. The key difference between these approaches is the explicit treatment of bug-detection failures, which are believed to be common [13], [14], [28], in site-occupancy models [14]–[16] (see Text S1). Logit-binomial GLMs yielded consistently small estimates of covariate effects and their variances, at times much smaller than would seem reasonable (Fig. 2). This was true both when modeling the results of single bug-searches (which mimic standard practice but yield unreliably low infestation indices) and when modeling the combined results of three consecutive bug-searches (which yield higher detection probabilities and hence infestation indices that are closer to site-occupancy estimates) (Figs. 2 and 3).

While a detailed analysis of these discrepancies is beyond the scope of the present paper, we stress that overly small coefficient estimates and overly narrow CIs may lead to flawed inferences about the ecology of the study organism. Most triatomine studies to date, including our own, have relied on single bug-searches and have ignored, at least formally, detection failures (but see refs. [13], [28]). We therefore suggest that some caution is required when interpreting the bulk of the triatomine population-ecology literature: important drivers of infestation may have been reported as having ‘small’ or ‘non-significant’ effects simply because those effects were underestimated, and non-important ones may have been reported as ‘significant’ simply because SEs were too small.

Furthermore, reliable infestation estimates are also critical for transmission-risk assessment and decision-making in the context of vector control-surveillance [41]. Mounting empirical evidence, such as that presented here and elsewhere [13], [41], shows how naïve indices based on active bug-searches are biased down, sometimes severely (Fig. 3, Text S1). In our study setting, naïve infestation indices from single bug-searches would have to be corrected upwards by a factor of ∼1.9 (1.4 to 2.2, depending on which bug-search is considered) to fairly reflect site-occupancy by *T. b. brasiliensis*. This suggests that pilot site-occupancy surveys could be conducted to derive ‘correction factors’ for the naïve infestation indices provided by routine vector surveillance; in our case, effective collaboration between academic and public health institutions made this possible. We however note that, due to varying infestation patterns and differences in bug-search team performance, the validity of such ‘correction factors’ would be limited to the specific spatial-temporal context in which they were derived. In our setting, many infestation foci went undetected during standard, single-visit bug-searches (Fig. 3); this can obviously help explain persistent dwelling re-infestation by *T. b. brasiliensis* in the study area (see also ref. [31]).

### Conclusions and outlook

Observational studies of animal ecology, including infectious disease ecology, involve two main sources of variation. One is related to the biological process under scrutiny, and this is usually the component of central interest [16]; what factors, for instance, modify the probability that individuals of a species will occupy a given habitat patch at a certain time-point? The other is related to sampling-process, or observation, uncertainty, and reflects the pervasive problem that the target organism may go undetected at sites that are, in reality, occupied [13]–[16], [42]. In the context of multiple hypothesis-testing, one further source of variance is model-selection uncertainty, which we incorporate by computing unconditional SEs for model-averaged 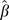
s [18], [26]. More generally, modeling complex biological systems with imperfect field data and simplified mathematical devices is intrinsically challenging [16], [18], [21]; even our best-fitting models will leave a fraction of the variance unexplained, and this matters when the conclusions can influence public health policy [14], [18], [28], [29].

Our results show how incorporating these sources of uncertainty into a robust analytical framework can further our understanding of the population ecology of a major disease vector. Based on our comparison of GLMs and site-occupancy models, we suggest that our main conclusions about (i) the importance of some key vertebrate hosts, (ii) the relatively small effects of ecotope structural traits, and (iii) the bearing of peridomestic vector colonies on house infestation risk are particularly reliable. Because we provide an explicit treatment of uncertainty, they are also more transparent than most previously published results. We anticipate that future research on triatomine ecology (and, in general, disease ecology [42]) will gradually incorporate methods and approaches similar to those we describe here. They can enhance our understanding of the drivers of site-occupancy by disease vectors, hosts, or pathogens [28], [29], [42], and, consequently, our ability to design and operate more effective control-surveillance systems.

## Supporting Information

Table S1 The complete set of site-occupancy models. The subset of models with ΣAkaike weights ≈0.95 is highlighted in blue, and ‘null’ occupancy models in pink. QAICc, quasi-AICc (AICc, Akaike information criterion corrected for sample size); ΔQAICc, difference in QAICc between each model and the lowest-QAICc (top-ranking) model; *w*_i_, Akaike model weight; Likelihood, likelihood of each model, given the data (or relative strength of evidence for each model); *k*, number of model parameters; Deviance, –2log-likelihood of each model. See main text for the definitions and values of covariates.
(XLSX)

Text S1 Model description and further possible sources of sampling-process heterogeneity.
(PDF)
