# Supplementary material for: Modeling Disease Vector Occurrence When Detection Is Imperfect II: Drivers of Site-Occupancy by Synanthropic Triatoma brasiliensis in the Brazilian Northeast
Source: PLoS Negl Trop Dis. 2014 May 8;8(5):e2861. doi: 10.1371/journal.pntd.0002861 (PMC4014420; doi:10.1371/journal.pntd.0002861)
Supplement: Text S1 — Model description and further possible sources of sampling-process heterogeneity. (PDF) [file pntd.0002861.s002.pdf]

Text S1

Model description and further possible sources of sampling-process heterogeneity

1. Dealing with imperfect detection: basic issues and model description

Suppose you are trying to determine the pattern of infestation of a series of discrete ecotopes (houses, corrals, henhouses, etc.) by a disease vector. In any realistic scenario, available methods for detecting the vector will all be imperfect – that is, some observed "absences" will in fact be *false-negative* results due to detection failures. Therefore, the observed fraction of ecotopes in which the vector was detected (the naïve "infestation index") will be biased down. Let *n* be the sample of ecotopes and *x* the ecotopes where vectors were detected; then, let *p* be the probability that a vector is detected in an ecotope during one search, given that the ecotope is infested (or "occupied") by the vector. The probability of detecting the vectors at least once after *s* searches in one ecotope is $p_s = 1 - (1 - p)^s$, and an estimator of the fraction of ecotopes that are occupied is $\widehat{\Psi} = \frac{x}{(n \times p_s)}$ (see ref. [S1]). There is an obvious correspondence between this estimator and the naïve infestation index ($\mathrm{II} = \frac{x}{n}$) routinely used in vector studies and vector control-surveillance systems (e.g., refs. [S2,S3]): both are equivalent *only* if $p = 1.0$, that is, if the sensitivity of the vector-detection method is 100%. Thus, seemingly simple naïve infestation indices implicitly make the strong assumption that the vectors are detected without error. Since this assumption is extremely unlikely to be met in any realistic situation, naïve infestation indices are all almost certainly biased down. This can confound our view of the vectors' ecology and hinder the development, implementation, management, and assessment of effective control-surveillance strategies.

One straightforward way of dealing with this pervasive problem is repeated ecotope sampling [S1,S4]. Repeatedly searching an ecotope yields a "detection history" consisting of a series of "0s" (non-detections) and "1s" (detections). For example, the results of searching an ecotope four times could be "0100", meaning that infestation was detected only during the second of four visits. If searches are made during a short time-period, so we can safely exclude ecotope-level extinction or colonization (i.e., assuming population 'closure'), we now know (i) that the ecotope was indeed infested and (ii) that we failed to detect the vector three times (we have three false-negative results). With the set of such detection histories derived from each ecotope in our sample, we can estimate the values of important parameters such as site-occupancy (Ψ) and detection (p) probabilities. One approach to do this is maximum-likelihood, where the target parameter values are those that maximize the probability of observing the data at hand under a given model. A very simplified example, taken from ref. [S4], follows.

Suppose that you have repeatedly searched a random sample of 10 ecotopes, with four visits to each ecotope, and got the following detection histories:

**Ecotope**	**Visit 1**	**Visit 2**	**Visit 3**	**Visit 4**	**Combined**
Ecotope 1	1	1	0	1	1
Ecotope 2	0	0	1	1	1
Ecotope 3	0	1	1	0	1
Ecotope 4	1	0	1	1	1
Ecotope 5	0	0	0	0	0
Ecotope 6	1	1	1	0	1
Ecotope 7	0	0	0	0	0
Ecotope 8	0	0	0	1	1
Ecotope 9	0	1	0	1	1
Ecotope 10	1	1	0	0	1
**Naïve infestation**					
Visit-specific	40%	50%	40%	50%	-
Cumulative	40%	60%	70%	80%	80%

1 = at least one vector was detected; 0 = no vectors were detected

Let p be the probability of observing infestation (represented by "1") in a visit to an infested ecotope; then, that of observing "0" is $1 - p$. The probability of observing the first detection history ("1101") would equal Ψ (the probability that the vector is present,

which we know must be true because it was detected) times the observed detection probabilities for each visit:

$$Pr(1101) = \Psi \times p \times p \times (1 - p) \times p = \Psi \times p^3 \times (1 - p).$$

The probability of observing the second detection history ("0011") is

$$Pr(0011) = \Psi \times (1 - p) \times (1 - p) \times p \times p = \Psi \times p^2 \times (1 - p)^2,$$

and so on for the remaining histories. The two "0000" histories, however, require a slightly more complex treatment, because they imply two mutually-exclusive possibilities: either the vectors (i) were present (Ψ) but went undetected even after four searches [$(1 – p)^4$] or (ii) were truly absent ($1 – \Psi$); therefore,

$$Pr(0000) = \Psi \times (1 - p)^4 + (1 - \Psi).$$

Given that each ecotope represents an independent observation, the joint probability of the complete dataset (the likelihood) is estimated by multiplying the probabilities for each ecotope:

$$Pr(\text{data}) = \prod Pr_i = [\Psi^8 \times p^{18} \times (1 - p)^{14}] \times [\Psi \times (1 - p)^4 + (1 - \Psi)]^2.$$

In this simple situation (a 'completely null' model without covariates) we have just two parameters to estimate: mean Ψ over ecotopes and mean p over ecotope-searches. Using the freely available program PRESENCE [S5], we can estimate the values that maximize the likelihood of the data (and their variances); in this case, $\hat{\Psi}$ = 0.839, SE = 0.137, and $\hat{p}$ = 0.537, SE = 0.098. Note that the occupancy estimate is ~4% higher than the naïve index of 0.8 observed after four visits – which, in addition, lacks a measure of uncertainty. Note also that the results of each single visit, when considered in isolation, yield badly biased infestation indices (0.4–0.5): many infestation foci go undetected (see also Fig. 3 of the main text). Unfortunately, the use of single-visit indices is standard practice in vector ecology studies and in vector control-surveillance program management [S2–S4,S6]. The estimate of p is a measure of the average sensitivity of

the vector-searching method, which in our example was ~54% (95%CI, ~35% to 72%) for each individual ecotope-search. We again emphasize that this is an extremely simplified example intended only as a basic illustration of the rationale of the method; interested readers can find an extensive and detailed treatment of this and other site-occupancy models in ref. [S1].

Using this approach, both key parameters (Ψ and *p*) can in addition be modeled as a function of covariates. For instance, we may be interested in finding out whether and to what extent infestation probabilities (Ψ) vary with the characteristics of ecotopes, among distinct localities, or as a consequence of a vector control intervention. Detection probabilities (*p*) may also be of interest, particularly when the performances of two or more bug-detection strategies (e.g., manual searches *vs*. vector-sensing devices, or different vector-sensing devices) are being compared [S4,S6]. Because both occupancy and detection probabilities are binomial variables, the natural link function is

$$\mathrm{logit}(\theta_i) = \ln\left(\frac{\theta_i}{(1-\theta_i)}\right) = \beta_0 + \beta_1 \times x_{i1} + \beta_2 \times x_{i2} + \cdots + \beta_C \times x_{iC},$$

where θ_i is the probability (or parameter) of interest for the ith sampling unit, β_0 is the intercept of the regression equation, and the rest of βs are the regression (slope) coefficients for covariates 1 to C [S1]. Because each estimate of Ψ, *p*, or β has an associated variance (which quantifies our uncertainty about the point estimate), we can compute confidence intervals that allow comparing the estimates; for example, we may ask whether infestation estimates are the same in different ecotope types, or to what extent detection probabilities change when a new vector-detection method is introduced. In the case of covariate effects, the analyses will yield a point estimate, positive or negative, of each β coefficient, as well as its variance or SE; the statistical significance of each effect can then be assessed by constructing appropriate confidence intervals and checking whether they encompass zero.

2. Testing further potential sources of sampling-process heterogeneity

The fact that goodness-of-fit testing detected moderate overdispersion suggested that there were further, un-modeled sources of sampling-process heterogeneity in addition to those considered in the assessment of 'null' models (see main text). Aiming to address this issue, we tested other factors that could possibly affect sampling outcomes, including covariates:

(i) Describing, for each bug-search and ecotope, the result of the previous bug-search;

(ii) Distinguishing the third bug search (performed after insecticide spraying) from the previous two;

(iii) Indexing whether any individual search was performed by the same team that performed the previous one (this happened in a few cases in searches 2/3); and

(iv) Indexing whether individual bug-searches were carried out with/without supervision from a member of the research team (this happened in some third visits).

As mentioned in the main text, none of these covariates improved model fit in terms of either QAICc or overdispersion (details not shown).

Supplementary references

S1. MacKenzie DI, Nichols JD, Royle JA, Pollock KH, Bailey LL, et al. (2006) Occupancy Estimation and Modeling: Inferring Patterns and Dynamics of Species Occurrence. San Diego: Elsevier Academic Press.

S2. World Health Organization (2002) Control of Chagas Disease: Second Report of the WHO Expert Committee. WHO Tech Rep Ser 905: i–vi 1–109.

S3. World Health Organization – TDR (2009) Dengue: Guidelines for Diagnosis, Treatment, Prevention and Control. Geneva: WHO/TDR.

S4. Abad-Franch F, Ferraz G (2010) "Didn't you see that bug…?" Investigating disease vector occurrence when detection is imperfect. Rev Soc Bras Med Trop 43 (Suppl. II): 31–34.

S5. Hines JE (2006) PRESENCE – Software to estimate patch occupancy and related parameters. USGS-PWRC. Available: http://www.mbr-pwrc.usgs.gov/software/presence.html.

S6. Rojas de Arias A, Abad-Franch F, Acosta N, López E, González N, et al. (2012) Post-control surveillance of *Triatoma infestans* and *Triatoma sordida* with chemically-baited sticky traps. PLoS Negl Trop Dis 6: e1822.
